# Acute lung allograft dysfunction predicts lung allograft failure: Construct validity of the 2026 International Society for Heart and Lung Transplantation spirometric definition

**DOI:** 10.1016/j.healun.2026.01.006

**Published:** 2026-05

**Authors:** John R. Greenland, Ciara M. Shaver, Krishna Pandya, Michael S. Mulligan, Michaela R. Anderson, Daniel R. Calabrese, Jason D. Christie, Lung Transplant Outcomes Group

**Affiliations:** a Department of Medicine, University of California San Francisco, San Francisco, CA; b San Francisco VA Health Care System, San Francisco, CA; c Departments of Medicine and Pathology, Microbiology, and Immunology, Vanderbilt University Medical Center, Nashville, TN; d Department of Medicine, University of Pennsylvania, Philadelphia, PA; e University of Washington, Seattle, WA.

**Keywords:** Lung transplantation, Acute lung allograft dysfunction (ALAD), Chronic lung allograft dysfunction (CLAD), Graft failure, Spirometry, Consensus validation

## Abstract

**INTRODUCTION:**

Acute lung allograft dysfunction (ALAD) has been proposed as a screening criterion to identify lung transplant recipients at increased risk for graft loss. ALAD was defined formally in a 2026 International Society for Heart and Lung Transplantation (ISHLT) Delphi consensus statement but has not been empirically evaluated. We sought to test the construct validity of the ISHLT ALAD definition by testing the hypothesis that ALAD is associated with increased risk of graft failure.

**METHODS:**

We included 86,885 spirometry measurements from 2,877 participants in the 8-center Lung Transplant Outcomes Group cohort. ALAD was defined as a ≥10% decline in FEV1 from the maximum value over the preceding 180 days and coded along with 90-day follow-up categories of *resolved*, *persistent*, *progressive*, and *rapidly progressive.* We assessed whether ALAD classifications diverged in predictions of time to death or re-transplantation in multivariable-adjusted time-dependent and landmark Cox proportional hazards models.

**RESULTS:**

ALAD had a prevalence of 15%. While resolution was seen in 40%, ALAD conferred a 4.93-fold graft failure hazard (95% CI 4.47–5.45, 7.4 years median follow-up), independent of concurrent chronic or baseline lung allograft dysfunction (CLAD or BLAD) status. Resolved ALAD was not associated with increased graft failure risk, but persistent, progressive, rapidly progressive, and no follow-up FEV1 ALAD categories were associated with increasing hazards for graft failure. A 10-percent threshold for FEV1 decline and a 180-day window to set the FEV1 baseline demonstrated optimal discrimination.

**CONCLUSION:**

The 2026 ISHLT spirometric ALAD definitions identify graft failure risk, supporting their relevance in clinical interventions and further research.

## Background

Survival and quality of life improvements from lung transplantation are limited by high rates of graft failure. To identify graft failure risk at a time when early therapeutic interventions might be effective, a syndrome of acute lung allograft dysfunction (ALAD) has been proposed. While the term ALAD has been used in the literature for over 10 years,^1^ the lack of a formal definition has resulted in inconsistencies in how it is operationalized.^2^ International Society for Heart and Lung Transplantation (ISHLT) sponsored a working group of international experts to formalize the definition of ALAD and provide guidance on its etiologies and management, with a Delphi voting method used to achieve consensus.^3^ As a spirometric screening criterion, ALAD was defined as a 10% decline in FEV1 from the maximum value over the past 3 to 6 months. The workgroup also formalized terminology for ALAD follow-up status at 90-days, including *recovered, persistent, progressive,* and *rapidly progressive*. While these terms achieved consensus, their associations with graft failure risk have not been established.

Chronic lung allograft dysfunction (CLAD) is a syndrome of persistent ≥20% decline in 1-sec forced expiratory volume (FEV1)^4,5^ that affects around 50% of lung transplant recipients by 5 years.^6,7^ However, by the time of definite CLAD diagnosis, graft failure pathology, including constrictive bronchiolitis and pleuroparenchymal fibroelastosis, may be too advanced for certain therapies to be effective.^4^ On a molecular level, longitudinal analyses comparing early versus definite CLAD airway tissue shows a shift from immune-related to airway remodeling transcriptional programs.^8^ At the same time, there is evidence that CLAD-associated gene expression changes appear prior to detection of a 20% FEV1 decline, even in the absence of acute rejection pathology.^9–11^ It is hoped that the ALAD criteria can identify allograft risk at a time when CLAD might be prevented.

A key area of debate in the ISHLT Delphi process for defining ALAD was what the optimal reference window for FEV1 maximum should be. Reflecting the “acute” nature of ALAD, some felt the maximum FEV1 value in the past 3 months should be used. Recognizing that graft dysfunction progresses at variable rates and there may be few measurements within a strict 3-month window, others argued for a 6-month window. Ultimately, both approaches achieved consensus, although there was greater agreement for the 3-month window. While there was consensus that ALAD could coexist with CLAD, there were also discussions about how these concepts might interact.

Here, we sought to test the concept validity of the new ISHLT ALAD definition by testing the hypothesis that ALAD is linked to increased risk of graft failure, defined by death or retransplant. We also assessed ALAD prevalence, its overlap with CLAD, and a key area of uncertainty in the definition: whether the preceding 3 or 6 months of data should be used to set the baseline from which a 10% decline is assessed.

## Materials and methods

### Cohort

We included participants from the Lung Transplant Outcomes Group (LTOG) multicenter, NIH-sponsored cohort study (NCT00552357), transplanted between 2004 and 2022. All participants provided written informed consent, and this study was approved by institutional review boards at participating centers. Participants without post-transplant pulmonary function testing or survival data were excluded. In cases where multiple FEV1 measurements were performed on the participant on the same day, only the first record was selected.

### Definitions

Spirometric definitions for ALAD were coded based on the 2026 ISHLT guidelines.^3^ Code in R format for assigning ALAD designations is included as Supplemental Code.

ALAD assignments were made for each available FEV1 measurement. ALAD was defined a ≥ 10% decline in FEV1 from a maximum value within the past 90 or 180 days, except where otherwise stated. If no FEV1 was measured in the prior 180 days, the most recent value was used. Follow-up classifications were determined based on the maximum FEV1 value within the subsequent 90 days after ALAD assessment (or 30 days for *rapidly progressive* ALAD). If no measure was available in 90 days, the next available value was used. Participants with no follow-up data were categorized separately as *no follow-up FEV1s*. *Recovered* ALAD indicated a follow-up FEV1 above the 10% FEV1 decline threshold. *Progressive* ALAD indicated an additional 10% decline from the FEV1 value in question. *Rapidly Progressive* ALAD was a subset of *Progressive* ALAD with a 10% decline in FEV1 within 30 days. *Persistent* ALAD indicated neither recovery nor progression.

Graft failure was assessed as the date of death or re-transplantation. CLAD was defined at the time of spirometry based on prior data as a 20% decline in FEV1 from a baseline of the best 2 values at least 3 weeks apart. *Possible* CLAD lasted less than 21 days, *probable* CLAD lasted less than 91 days, while *definite* CLAD persisted for at least 91 days.^4^ The censor date was that of the most recent contact prior to the study end date in May 2025. Spirometry after re-transplantation was not included.

Baseline lung allograft dysfunction (BLAD) was defined in recipients with > 1-year of post-transplant spirometry as a baseline FEV1 less than the lower limit of normal, as predicted from recipient information using race-neutral Global Lung Function Initiative equations.^12,13^ Missing lower limit of normal data were imputed by random forest model.^14^

### Analysis

To assess the association between ALAD designation and graft failure risk, we employed Cox proportional hazards models. The primary exposure was ALAD status, modeled as a time-dependent binary variable indicating whether the subject was in the state of ALAD at each spirometry time point. ALAD exposure may have been interrupted if the participant experienced multiple episodes of ALAD. Because survival models included multiple time intervals for each participant, corresponding to repeat spirometry assessments where ALAD status may change, robust variance estimates clustered by participant were used to account for within participant correlation. As an exploratory analysis, we also examined a cumulative time-dependent model, where the predictor was the count of discrete episodes of ALAD. All models were adjusted for baseline characteristics of recipient age and sex, transplant indication, transplant procedure, and recipient race/ethnicity, as coded in Table 1. Participating site was included as a stratification variable. Differences in ALAD outcomes across centers were assessed using a permutation test of the variance component for the center-by-ALAD interaction (random effect) in a mixed-effects Cox proportional hazards model. Center labels were permuted 200 times at the participant ID level, and the model was refit for each permutation to define a null distribution.^15^ Between-center differences in baseline hazard were similarly assessed.

To test the construct validity of 90-day follow-up definitions, we evaluated whether ALAD recovery trajectories, categorized as *recovered*, *persistent*, *progressive*, or *rapidly progressive*, were associated with escalating risks of subsequent death or retransplantation. Using a time-dependent Cox model, the exposure variable was the 90-day follow-up category, coded as a factor starting 90 days after ALAD ascertainment to ensure independence between the exposure and the outcome windows. Baseline characteristics were included as covariates, and the outcome was time to graft failure.

Model fit was assessed using Akaike’s Information Criterion (AIC), with Akaike weight percentages indicating 0% to 100% relative support for a given model amongst alternatives.^16^ Model discrimination was assessed based on the concordance metric. A concordance of 100% would indicate that the survival times for those with ALAD were all less than survival times for those who didn’t meet ALAD criteria. Concordance is the equivalent of the area under the receiver operating curve for time dependent models. Differences in concordance were assessed by bootstrapping.

While time-dependent models were used to assess the overall association between ALAD status and graft outcomes, complementary landmark models examined whether the strength of this association varied across post-transplant time periods, recognizing that early and late ALAD may reflect different underlying processes. For landmark Cox proportional hazards models, we assessed each day in the first 3.5 or 5 years post-transplant, shown as the x-axis on Figures 2 and 4B, respectively. Each participant contributed only one observation per landmark time, using the ALAD classification closest to a given time point within a maximum window of 45 days in either direction. Models were adjusted for covariates and stratified as above. Graft failure time was assessed as time from ALAD assignment or from 90-day follow-up categorization to death or retransplant.

Longitudinal prevalence was calculated as days with ALAD divided by days under observation. We selected this prevalence metric because it was less dependent on the frequency of spirometry and did not require distinguishing contiguous ALAD events.^17^ A logistic generalized-estimating equation regression model was used to assess the association between ALAD status and participant characteristics.

ALAD and CLAD co-occurrence was assessed by χ^2^-test and plotting of the standardized observed-expected residual values. To avoid bias from repeat measures in these analyses, 1 spirometry was sampled at random per participant, and the median from 1,000 replicates is shown (Figure 5). BLAD status was computed for each spirometry value and assessed as a time-dependent covariate or stratification variable in survival models. “Not BLAD” was the reference compared with “undefined” (< 1-year) or “BLAD.”

Analyses were performed in R (Version 3.2, R Foundation for Statistical Computing, Viena Austria), using the *survival, coxme, survminer, ggplot2, geeglm, tableone, dplyr, magrittr, tidyr, doParallel, randomforest,* and *foreach* libraries.

## Results

**Figure 1:**
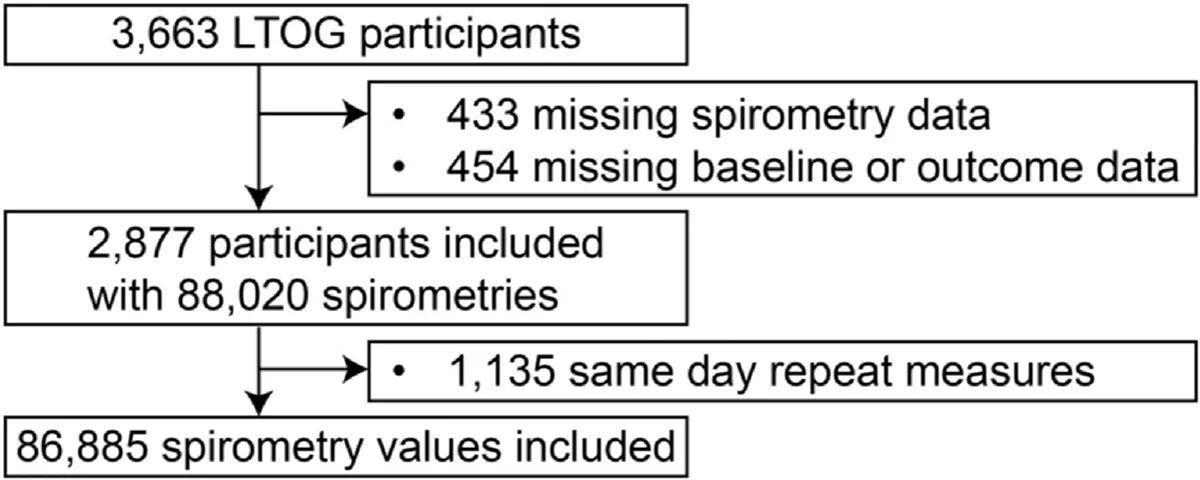
CONSORT flow diagram of study participants.

Of the 3,663 participants in the LTOG cohort, 433 were excluded because of missing spirometry data and 454 were excluded because of missing follow-up data, resulting in a total cohort of 2877 participants (Figure 1). Characteristics of the participants are shown in Table 1.

### ALAD frequency and 90-day follow-up status

As shown in Table 2, from the 86,885 unique FEV1 measurements in this cohort, ALAD criteria were met for 22% of spirometry values. Within the study follow-up period, 89% of participants had at least 1 episode of ALAD. ALAD was more common in recipients who were men (OR 1.17, 95% CI 1.08–1.26), Black/African American (HR 1.16, 95% CI 1.03–1.32), had obstructive indications (HR 1.18, 95% CI 1.08–1.29) or single lung transplants (HR 1.11, 95% CI 1.01–1.21), and at some centers.

Most (60%) of spirometry measurements meeting criteria for ALAD did not recover at 90 days. From those studies classified as ALAD, ALAD persisted in 45% and progressed in 10%. For 5% of studies with ALAD, there were no follow-up FEV1 measurements. The first value for each participant had no prior FEV1s, leading to 3% of studies being uncategorized. While the window for the FEV1 baseline spanned 180 days, the maximum prior FEV1 value was observed at a median of 91 days prior (IQR 42–135).

### ALAD is associated with graft failure

Consistent with our overall hypothesis, ALAD classification was associated with a 4.93-fold risk of graft failure (95% CI 4.47–5.45, *p* < 0.0001) from the time of FEV1 measurement. We then assessed graft failure risk as a function of follow-up status. Table 3 shows the adjusted risk of graft failure from the time of ALAD follow-up classification, 90 days later. Recovery from ALAD conferred no increased risk of graft failure, but ALAD that did not recover was associated with a 5.71-fold increased risk of graft failure (95% CI 5.14–6.34, *p* < 0.0001) versus no ALAD. We observed a progressive increase in graft failure risk across *persistent*, *progressive*, *rapidly progressive*, and *no follow-up FEV1* strata.

These follow-up categories added meaningful information: compared with a model including all 5 ALAD categories, merging *rapidly progressive* and *progressive* categories resulted in a somewhat worse fit (ΔAIC = 5.6, W_merged_ = 6%). A model assessing all unrecovered ALAD versus recovered or no ALAD resulted in substantially worse fit versus the 5-category model (ΔAIC = 411, W_merged_ = 0%). ALAD was meaningful in the subset of single lung transplant recipients, but the hazard ratio decreased to 3.56 (95% CI 2.96–4.29), suggesting that transplant type modifies the association between ALAD and outcomes (*P*_interaction_ < 0.001). Compared to a model using the cumulative count of discrete ALAD episodes as a predictor, the binary ALAD model fit the data substantially better (ΔAIC = 789, W_binary_ = 100%, W_cumulative_ = 0%), suggesting that recurrent ALAD episodes do not confer greater risk versus the ALAD state itself.

**Figure 2:**
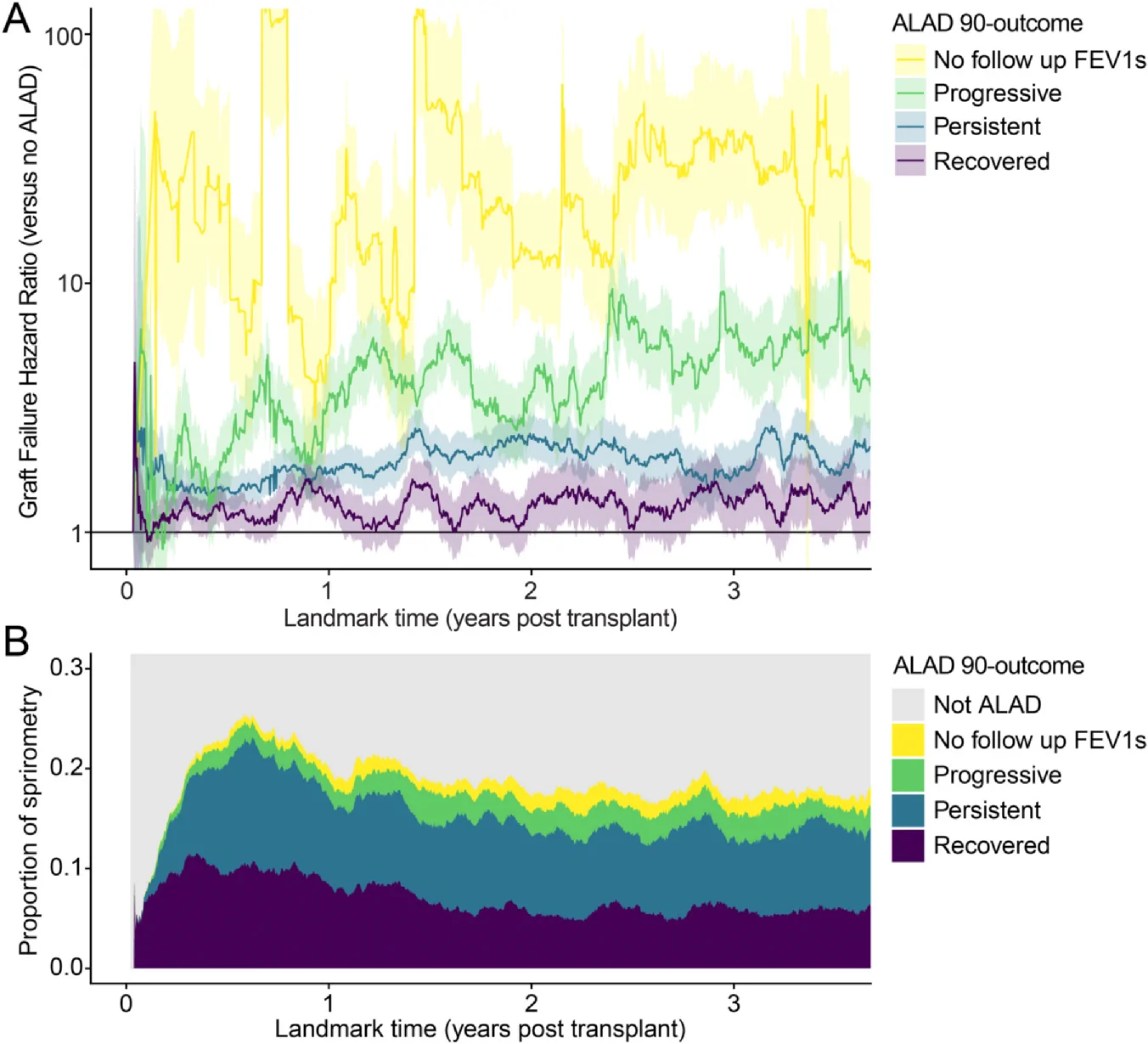
Comparison of graft failure risk according to ALAD 90-day follow-up categories. **(A)** Landmark analyses showing the adjusted hazard ratio for graft failure (death or retransplant) stratified by ALAD categorization. For each landmark time on the x-axis, ALAD was assessed based on the spirometry closest to that timepoint for each individual. The hazard ratio for graft failure from the time of 90-day follow-up is then shown as a function of the time point where ALAD was assessed. (Individuals are only included once in each hazard ratio calculation with their ALAD status at the landmark time.) Ribbons denote 95% confidence intervals. **(B)** The proportions in each ALAD follow up category are shown for each landmark timepoint (x-axis) by area plot. ALAD, acute lung allograft dysfunction.

To understand the impact of ALAD classifications over time post-transplant and avoid potential complexities in interpreting time-dependent covariate coefficients, we performed landmark analyses over the first 3 years post-transplant. As shown in Figure 2A, separation between outcomes for *recovered, persistent*, *progressive*, *rapidly progressive*, and *no follow-up FEV1* strata emerged around 90 days post-transplant and persisted through 3 years. The absence of follow-up FEV1 data conferred a substantial increase in risk of graft loss. For studies without follow-up spirometry, the median survival was 74 days (95% CI 69–83 days). From Figure 2B, ALAD prevalence peaked around 7 months, and recovery became less common over time. Variation in prevalence and hazard ratios over time may reflect sampling heterogeneity or contributions of pulmonary function testing done *for cause* outside of normal surveillance intervals.

We asked whether there was variation in outcomes across centers, which could reflect center-specific differences in approaches to identifying and treating ALAD, patient populations, or ALAD etiologies. A mixed effects Cox model showed both statistically significant interactions between ALAD status and center (*p* < 0.01) and differences in baseline hazards across centers (*p* < 0.01). The variation in hazards by site is shown in Supplemental Figure 1. These data suggest important heterogeneity in ALAD outcomes across center.

### A 10% FEV1 threshold is appropriate

**Figure 3:**
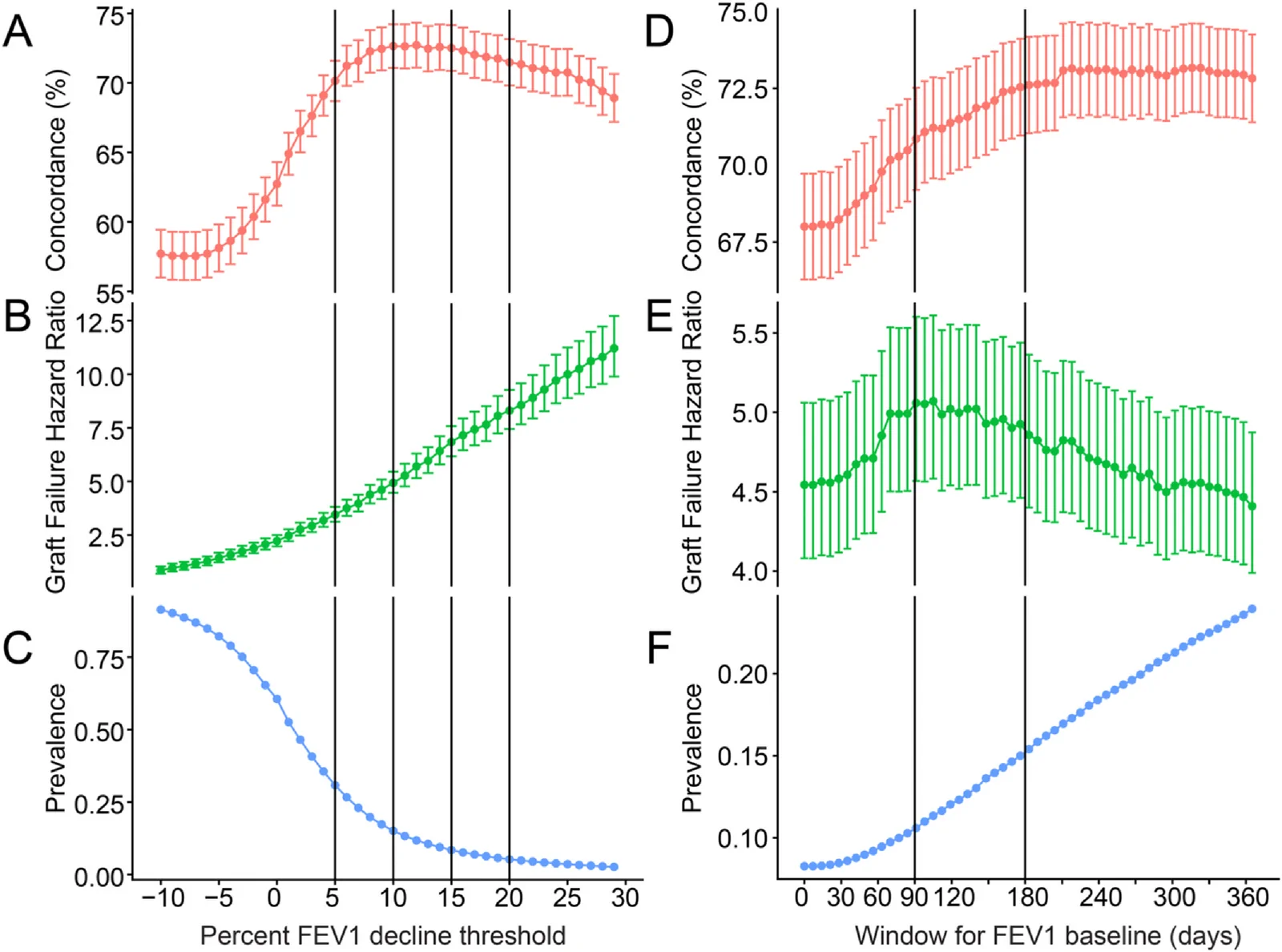
Sensitivity analysis for FEV1 threshold and baseline windows. (**A-C**) demonstrate the effect of varying the threshold for identifying ALAD, typically at a ≥10% decline in FEV1, from a 10% gain to a ≥30% decline. (**D-F**) assess the impact of varying the number of days prior to the spirometry to set the baseline of FEV1. A window of 0 days indicates that the most recent prior FEV1 would be used to set the baseline, while a window of 180-days would use a maximum value over the past 180 days. (**A&D**) Concordance measures show how changing ALAD definitions impact time to graft failure model discrimination. Concordance is the probability, given a random pair of recipients, that the 1 meeting criteria for ALAD would have an earlier time to graft failure, with 1 being a perfect classifier and 0.5 being expected by chance. (**B&E**) Graft failure (death or retransplant) risk is show across definitions, with ALAD status as a time-dependent covariate in a Cox proportional hazards model adjusted for baseline characteristics. Error bars indicate 95% confidence intervals. (**C&F**) The impact of changing ALAD definitions on its prevalence is shown, with a value of 1 indicating that every recipient was always in the state of ALAD. Prevalence is calculated as the time with ALAD over time under observation. ALAD, acute lung allograft dysfunction; FEV1, 1-sec forced expiratory volume.

The 10% threshold for FEV1 decline is based on the understanding of normal biological variability and measurement error in spirometry.^18^ However, its validity in the context of diagnosing lung allograft dysfunction has not been demonstrated. We assessed the impact of the threshold for FEV1 decline with respect to optimal discrimination of and association with graft failure risk. As shown in Figure 3A, concordance (discrimination) improved sharply for FEV1 thresholds up to 5% and was greatest in the range of 9%-15%. The maximum value was at 12%, with a concordance of 72.7% (95% CI 71.1%-74.3%). The hazard ratio for graft failure (Figure 3B) increased with greater threshold values, but this was associated with a decline in prevalence (Figure 3C). Overall, the threshold of 10% had a strong concordance (72.7%, 95% CI 71.1%-74.2%), strong association (HR 4.93, 95% 4.47–5.45), and reasonable prevalence (15.1%, corresponding to 22.7% of included spirometry values).

### A 6-month window for setting the maximum FEV1 baseline is optimal

We assessed the impact of different FEV1 baseline windows on the discrimination and association with ALAD. As shown in Figure 3D, there was increasing concordance with longer windows, with a maximum value of 73.2% (95% CI 71.7%-74.6%) at 316 days. The degree of association peaked at 140 days with a HR for graft failure 5.02 (95% CI 4.54–5.55, Figure 3E). There was a steady increase in ALAD prevalence as the window increased (Figure 3F). Compared with a 90-day window, 180 days captured significantly more individuals at increased risk of poor outcomes. Compared with a prevalence of 10.5% with a 90-day window, a 180-day window had a prevalence of 15.1% (43% increase). There was no detectable difference in graft failure hazard rate: The 180-day hazard ratio was 4.93 (95% CI 4.47–5.45), while the 90-day HR was 5.05 (95% CI 4.56–5.60). There was an increase in concordance from 70.8% (95% CI 69.1%-72.5%) with a 90-day window to 72.7% (95% CI 71.1%-74.2%) with a 180-day window (*p*-value for difference ≤0.001). Accordingly, found evidence of a superior fit for the model with a 180-day window, compared was a 90-day window model (ΔAIC = 93.5, W_90 day_ = 0%, W180 day = 100%).

All spirometry values meeting ALAD criteria with a 3-month window would meet criteria using a 6-month window, because looking back further can only increase the maximum FEV1. We compared participants who met ALAD criteria only when using the 6-month window (representing a “slow-burn” decline in lung function) and those who met criteria under both the 3- and 6-month definitions, with not ALAD as the reference group. In a time-dependent Cox model, ALAD detectable only by the 6-month window was significantly associated with graft failure compared with not ALAD (HR 3.25, 95% CI 2.73–3.87), although this association was weaker than for ALAD detected by both the 3- and 6-month windows (HR 5.65, 95% CI 5.07–6.27). These findings suggest that ALAD cases identifiable only with a longer window still confer elevated graft-failure risk, although to a lesser extent than those with more rapid decline detected by both windows.

**Figure 4:**
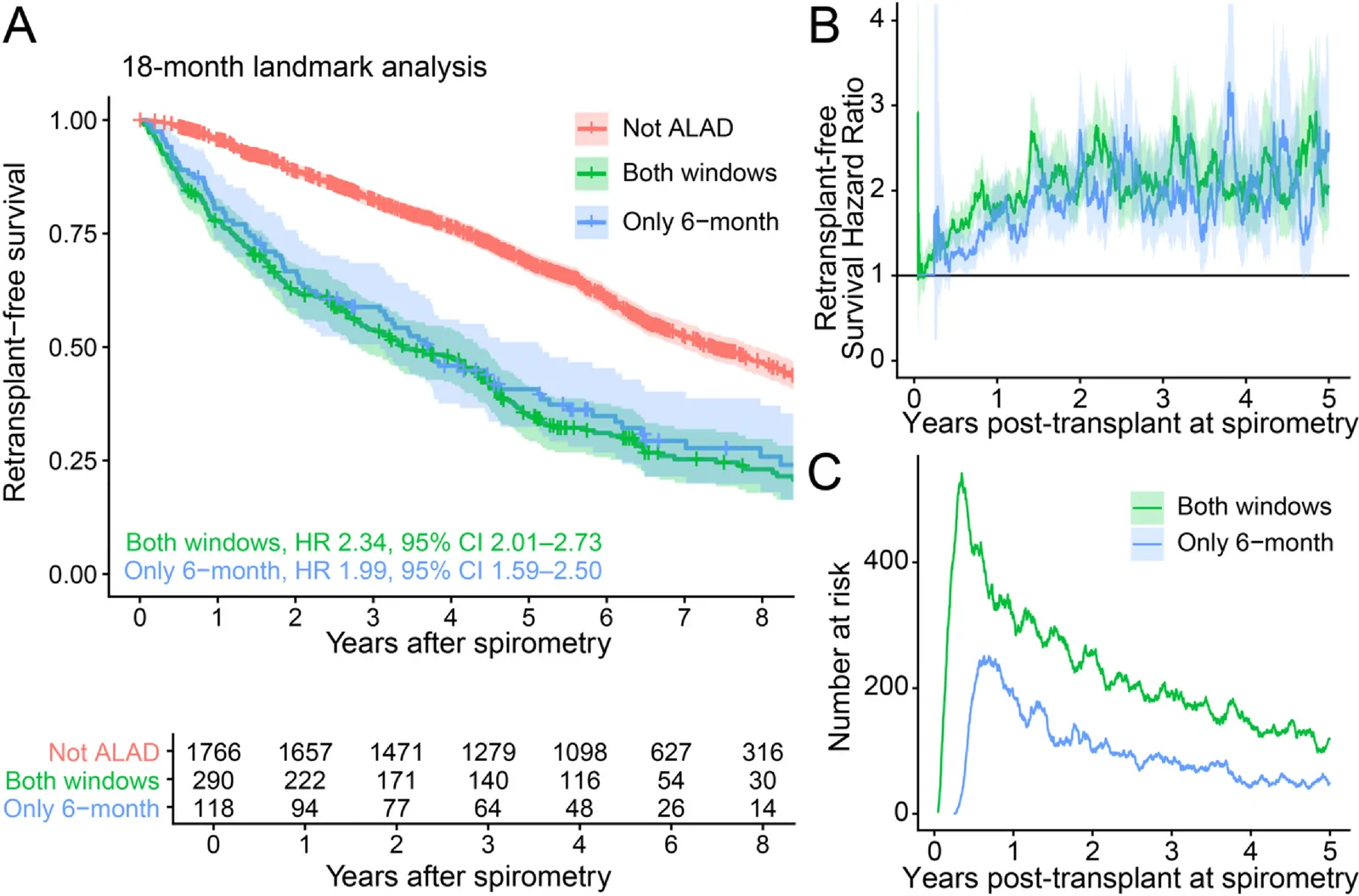
Landmark analysis comparing ALAD defined using 3 and 6 months baseline windows. ALAD was defined as a ≥10% decline in FEV1 from the maximum value over the past 3 or 6 months. Green indicates individuals that met criteria with the 3-month window (and thus would meet both definitions), while blue indicates the additional individuals who would be identified using a 6-month window but not the 3-month window. **(A)** shows a Kaplan–Meier plot of freedom from graft failure versus time from spirometry in a representative 18-month landmark analysis, where the closest spirometry to 18 months post-transplant for each individual was used as the predictor. Extending this analysis across all possible landmark times in the first 5 years, **(B)** shows the hazard ratios for graft failure as a function of the time post-transplant when the landmark spirometry value was set. Ribbons indicate 95% confidence intervals. **(C)** The number of participants at risk in these landmark analyses is shown as a function of spirometry date post-transplant for recipients meeting the 3-month ALAD criteria (which is sufficient for the 6-month definition as well, green) versus the additional participants who would only be identified using a 6-month FEV1 window (blue). ALAD, acute lung allograft dysfunction; FEV1, 1-sec forced expiratory volume.

We next performed complementary analyses of 3- and 6-month FEV1 baseline windows using landmark analyses. Figure 4A shows a representative landmark analysis of recipients using the closest spirometry value to 18 months post-transplant. Those with a slower decline in lung function that only met the 6-month ALAD criteria (but not at the 3-month window) had overlapping outcomes with those meeting ALAD definitions using the 3-month window. However, using the 6-month window increased the at-risk population by 41%. As shown in Figure 4B, looking across a range of landmark analyses, the hazard ratios for the 2 FEV1 reference windows become comparable at around 1 year post transplant. Consistent with Figure 2B, the risk associated with ALAD appears to increase over time and was less in the first post-transplant year. Figure 4C demonstrates that expanding the baseline window to 180 days would increase the number of at-risk individuals identified at any time by almost half.

### ALAD is applicable to individuals with CLAD and BLAD

**Figure 5:**
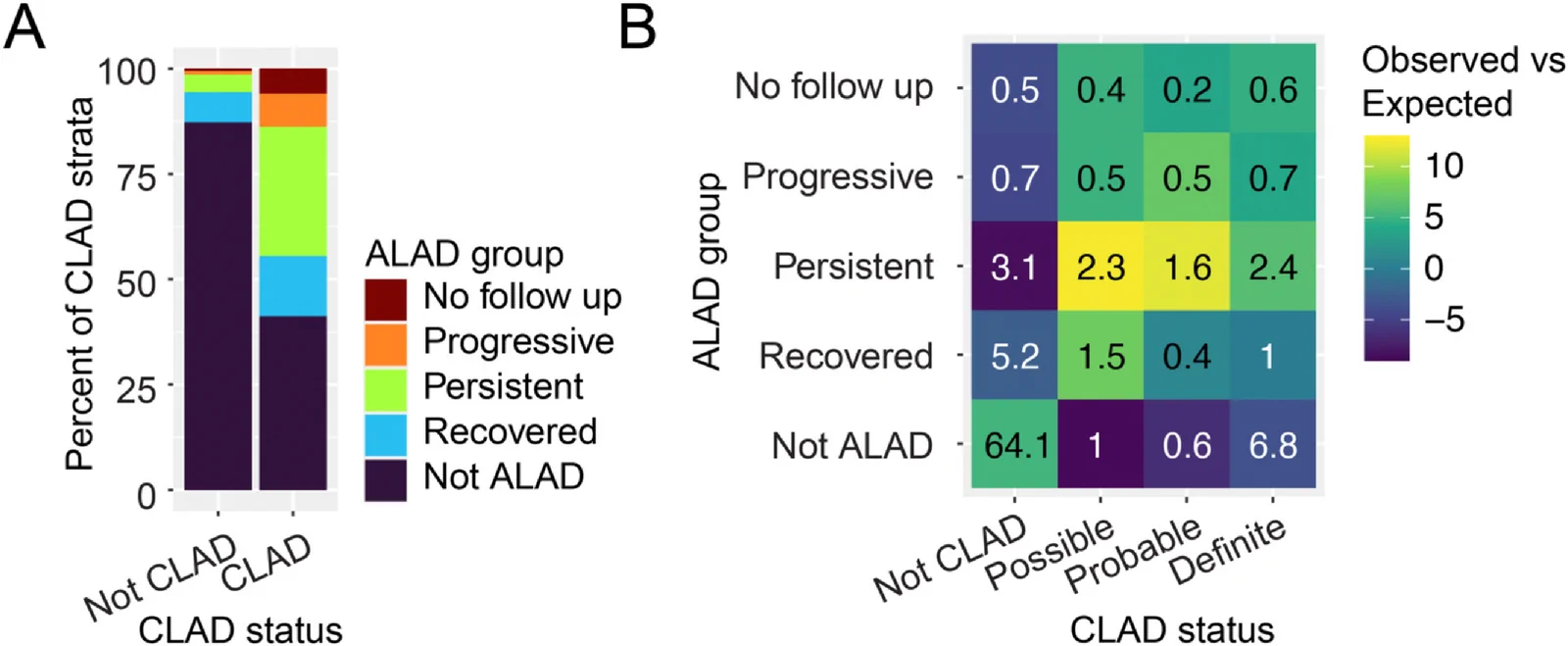
Co-occurrence of ALAD and CLAD. To understand the overlap between CLAD definitions, we determined the percentage of participants in each combination of ALAD and CLAD categories. (**A**) The percentage of ALAD 90-day follow-up categories are shown stratified by CLAD status (of any duration) for each spirometry. (**B**) Co-occurrence matrix of ALAD and CLAD classifications. The numbers indicate the percentage of spirometry results in each ALAD and CLAD category (of all spirometry results in the study). Colors indicate the standardized residual for a χ^2^-test, with yellow indicating that the pairing was more frequently observed than would be expected. To account for repeat measures, 1 spirometry per participant was randomly selected, and the median of 1,000 replicates is shown. ALAD, acute lung allograft dysfunction; CLAD, chronic lung allograft dysfunction.

We assessed the overlap between ALAD and CLAD definitions. Despite alignment in ALAD and CLAD categories, the overlap was incomplete, reflecting the moving baseline and transient nature of ALAD. Considering CLAD of any duration, ALAD was concurrent 58% of the time, while spirometry values without CLAD had ALAD only 13% of the time (Figure 5A). We observed 64.1% of spirometry values were classified as neither CLAD nor ALAD, which was greater than expected by chance. Persistent ALAD was enriched within the CLAD groups, particularly the possible and probable CLAD designations. Possible CLAD was linked to recovered ALAD (Figure 5B).

For the prediction of graft failure, we did not identify an interaction between ALAD and definite CLAD (*p* = 0.25). In the subset of individuals with CLAD, ALAD was associated with a 3.52 (95% CI 3.02–4.11, *p* < 0.0001)-fold increase in the graft failure risk. For those without CLAD, the hazard ratio was 4.29 (95% CI 3.72–4.94, *p* < 0.0001). Thus, while there was some overlap between ALAD and CLAD, ALAD was a risk factor for graft failure regardless of CLAD status.

We examined the effect of adding BLAD as a covariate in retransplant-free survival models. Both BLAD and ALAD were independent predictors of graft failure, with hazard ratios of 2.49 (95% CI 1.91–3.24) and 4.89 (95% CI 4.43–5.40), respectively. In a stratified analysis, the ALAD association was similar in recipients with BLAD (HR 4.90, 95% CI 4.06–5.90) versus not BLAD (HR 5.26, 95% CI 4.61–6.00). While BLAD was associated with worse outcomes, ALAD prognostic associations were independent of BLAD status.

## Discussion

Here we assessed the construct validity for the 2026 ISHLT consensus definitions of ALAD in a large, multicenter lung transplant cohort with extensive follow-up. We found that ALAD is associated with a substantial increase in graft failure risk. Sensitivity analyses support the definition of ALAD as a 10% decline in FEV1 from the maximum value over the preceding 180 days. While a window of 90 days has a strong association with graft failure, this range results in a substantial proportion of patients being misclassified as low-risk and significantly decreases model discrimination and goodness of fit. Our findings also support the proposed 90-day ALAD follow-up classifications, in that there was no increase in risk for graft failure for those with *recovered* ALAD but an increasing risk of graft failure across the categories of *persistent*, *progressive*, and *rapidly progressive* ALAD. These categories were associated with increases in model fit, although the subcategorization of *progressive* versus *rapidly progressive* provided only marginal additional information. While only 40% of spirometry results met criteria for *recovered* ALAD, even stabilization of lung function decline provides some reassurance.

ALAD may have advantages over BOS-0p, which relied on fixed a FEV_1_ baseline and a FEF_25%-75%_ criteria that had poor performance characteristics.^19^ By contrast, ALAD has a dynamic baseline based on the prior 3–6 months of data, does not require a 3-week confirmation measurement, and focuses solely on acute FEV1 declines, which may allow earlier identification of at-risk allografts and maintain prognostic significance even in the presence of CLAD or BLAD. Indeed, we observed that the association of ALAD with graft failure was equally important for participants with concurrent CLAD or BLAD. However, an important caveat is that we lacked data to exclude non-CLAD causes of FEV1 decline in this cohort, and so the results might vary if CLAD were adjudicated per ISHLT recommendations. Persistent or progressive ALAD beginning in the context of CLAD may overlap substantially with “progressive CLAD.” However, we propose maintaining ALAD as an independent designation, as it may have distinct molecular mechanisms and treatment implications.^8^ ALAD within CLAD may reflect an opportunity for intervention that could be missed if otherwise dismissed as progressive CLAD.

Spirometry data here were collected for clinical purposes and were likely acted on. As detailed in the ISHLT ALAD consensus document, management of ALAD may include additional diagnostic testing, such as imaging or bronchoscopy, and therapies targeted at the causes of ALAD, including acute cellular or antibody-mediated rejection, other forms of alloimmune injury, and non-immune injury.^2,3^ The present data do not assess these management strategies, but observed differences across centers could indicate that some management strategies are more effective than others. Timely interventions to address acute declines in pulmonary function might mitigate the severity of outcomes. Conversely, the risk of ALAD in the absence of any therapy might be greater than what was observed here.

While we focus on the spirometric definition of ALAD here, some recipients may meet clinical ALAD criteria based on hypoxemia, dyspnea, CT changes or other signs or symptoms, or hospitalization for respiratory failure. In some cases, recipients with ALAD may be too ill to undergo pulmonary function testing.^20^ The construct validity of clinical ALAD in the absence of spirometric changes remains to be determined. We used landmark analyses to complement time-dependent Cox models and to get a sense of how the construct validity of ALAD changes over time post-transplant. Interestingly, the significance of ALAD increased over the first 2 years after transplantation, which may reflect increasing stability in spirometry over time and that graft failure pathology is an indolent process that develops over years.

Graft failure risk is one important metric for construct validity, but there are other important potential associations to be investigated, such as the links with rejection and infection findings on bronchoscopy or patient-reported quality of life and physical function. Unfortunately, these data are not yet available within the LTOG cohort. While there are some data linking changes of early CLAD or ALAD to molecular changes in the airway brush transcriptome^8,11,21^ and the release of donor-derived cell-free DNA,^22,23^ these have not been prospectively addressed using the current definitions.

In summary, we found a strong association between ALAD and graft failure in a large, multicenter cohort, providing construct validity for the 2026 ISHLT definition. ALAD may be an important clinical and research designation for identifying lung allografts at risk of graft failure who may benefit from early intervention.

## Supplementary Material

1

2 Code
alad.fev1.bl.R contains code for calculating ALAD status from a table of study IDs, FEV1 values, and post-transplant dates. GitHub link to code: https://github.com/UCSFGreenlandLab/ALAD
During the preparation of this work, the authors used OpenEvidence and UCSF Versa (running Anthropic’s Claude Sonnet 4 and Microsoft’s Azure OpenAI GPT models) to perform literature reviews, draft code, and improve readability. After using these tools, the authors reviewed and edited the content as needed and take full responsibility for the content of the published article.

## Figures and Tables

**Table 1:** Baseline Characteristics

Characteristic	*N*	%/[IQR]

*N*	2,877	
Spirometry measurements, median [IQR]	27	[18–40]
Recipient age, median [IQR]	60	[50–66]
Male recipient, *N* (%)	1,625	56.5
Transplant indication, *N* (%)		
A-Obstructive	727	25.3
B-Pulmonary vascular	124	4.3
C-Bronchiectasis	421	14.6
D-Restrictive	1,540	53.5
Retransplant/Other	65	2.3
Transplant procedure, *N* (%)		
Double	2,120	73.7
Single	648	22.5
Multiorgan/lobar	109	3.8
Recipient race/ethnicity, *N* (%)		
White/Caucasian	2,310	80.3
Black/African American	233	8.1
Hispanic/Latino	187	6.5
Asian/Asian American	89	3.1
Other	58	2.0
Site, *N* (%)		
Columbia	338	11.7
Duke	507	17.6
Johns Hopkins	193	6.7
Indiana	187	6.5
University of Pennsylvania	697	24.2
Pittsburgh	269	9.4
Stanford	333	11.6
University California San Francisco	353	12.3

Abbreviations: *N*, number; IQR, interquartile range.

**Table 2:** ALAD Frequency and 90-Day Follow-up Status

	*N*	% of total	% of ALAD

No prior FEV_1_ measurement	2,877	3%	
Not ALAD	64,858	75%	
ALAD	19,190	22%	
Recovered	7,705	9%	40%
Persistent	8,686	10%	45%
Progressive	1,293	1%	7%
Rapidly progressive	567	1%	3%
No follow-up FEV_1_	899	1%	5%
Baseline days prior, median [IQR]	91 [42, 135]		

ALAD was classified for each spirometry measurement using a 180-day FEV1 baseline window and a 10% FEV1 decline threshold. Participants contributed multiple measurements.

Abbreviations: ALAD, acute lung allograft dysfunction; FEV_1_, 1-sec forced expiratory volume; IQR, interquartile range.

**Table 3:** Association Between ALAD 90-Day Follow-up Category and Subsequent Graft Failure Risk

	Hazard Ratio^a^	95% Confidence Interval	*p*-value

Recovered	0.84	0.62–1.12	0.23
Persistent	2.36	1.99–2.79	< 2·10^−16^
Progressive	6.84	5.63–8.30	< 2·10^−16^
Rapidly Progressive	13.9	8.98–21.5	< 2·10^−16^
No follow-up FEV1s	17.6	15.3–20.3	< 2·10^−16^

a A multivariable time-dependent Cox proportional hazards model included covariates of recipient age, sex, race/ethnicity, transplant indication, and transplant procedure; with “Not ALAD” as the reference group.

## Appendix A. Supplementary data

Supplementary data associated with this article can be found in the online version at doi:10.1016/j.healun.2026.01.006.

## Abbreviations:

AIC: Akaike’s information criterion
ALAD: acute lung allograft dysfunction
BLAD: baseline lung allograft dysfunction
CI: confidence interval
CLAD: chronic lung allograft dysfunction
FEV1: forced expiratory volume in 1-sec
HR: hazard ratio
IQR: interquartile range
ISHLT: International Society for Heart and Lung Transplantation
LTOG: Lung Transplant Outcomes Group
