# Supplementary material for: Acute lung allograft dysfunction predicts lung allograft failure: Construct validity of the 2026 International Society for Heart and Lung Transplantation spirometric definition
Source: J Heart Lung Transplant. Author manuscript; Available in PMC 2026 Sep 8. (PMC13552345; doi:10.1016/j.healun.2026.01.006)
Supplement: Supplementary file 1 [file NIHMS2189662-supplement-1.docx]

**Supplemental Figure 1. Variation in ALAD outcomes by LTOG center**

Graft failure (death or retransplantation) hazard ratios are shown with 95% confidence intervals for adjusted time-dependent Cox proportional hazards models for ALAD strata of recovered, persistent, progressive, rapidly progressive, and no follow up FEV1, as well as any ALAD, with Not ALAD as the reference group. Centers are shown on the y-axis, with Overall referring to the outcomes for the entire cohort. For the predictor of any ALAD, a statistically-significant interaction between the center variable and ALAD is denoted as “*” for P<0.05 and “.” for P<0.1.

**Supplemental Table 1. LTOG Investigators**

| **Site** | **Principal Investigator** | **Co-Investigator(s)** | **Study Coordinator(s)** |
| --- | --- | --- | --- |
| University of Pennsylvania | Jason D. Christie, M.D. | Ed Cantu, M.D.  Joshua Diamond, M.D.  Michaela Anderson, M.D.  Michael Shashaty, M.D. | Michelle Oyster  Melanie Brown  Melanie Rushefski  Laurel Kalman  Sheil Desai  Abigail Faust  Brian McCauley |
| Columbia University | David J. Lederer, M.D.  Luke Benvenuto, M.D. | Joshua Sonnett, M.D.  Selim Arcasoy, M.D. | Wendy Gonzalez  Onumaraekwu Opara  Shreena Patel  Anna Miller |
| University of Alabama at Birmingham* | Keith M. Wille, M.D. | Enrique Diaz-Guzman, M.D. | Mallory Halpin  Lanier O’Hare |
| Stanford University | Ann Weinacker, M.D.  Gundeep S Dhillon, M.D. | David Weill, M.D. | Tal Alfasi  Gowri Vasudevan |
| Johns Hopkins University | Jonathan Orens, M.D.  Pali Shah, M.D. | Christian Merlo, M.D.  Leann Silhann, M.D.  Ashish Shah, M.D. | Joby Matthew  Matt Kelley |
| University of Michigan* | Vibha Lama, M.D.  Michael Combs, M.D. |  | Mary Maliarik  Lisa Pollack  Krystyna Edgell |
| University of Pittsburgh | John McDyer, M.D. | Chadi Hage, M.D. | Brianna Hewitt  Swati Gulati  Vera Iouchmanov  Ritchie Koshy  Robin Burke |
| Duke University | Laurie Snyder, M.D.  Scott Palmer, M.D. | Matt Hartwig, M.D. | Allie Frear  Erika Bush  Brittany McDowell  Courtney Frankel  Alexis Paterson |
| University of Chicago* | Ed Garrity, M.D. | Chris Wigfield, M.D.  P.J. McShane, M.D. |  |
| Indiana University | Mrunal Patel, M.D. | David Roe, M.D.  Michael Duncan, M.D. | Jean Nash  Srdjan Kurbalija |
| University of California San Francisco | Jonathan Singer, M.D. | Carolyn Calfee, M.D.  Jasleen Kukreja, M.D.  John Greenland, M.D., Ph.D. | Legna Betancourt  Paige Gambetta  Hunter Scott |
| Vanderbilt University* | Lorraine Ware, M.D. |  |  |

* PFT data were not available from these centers.
